# Fertility sparing approach as the standard of care in young patients with immature teratomas

**DOI:** 10.4274/jtgga.2016.0090

**Published:** 2017-03-01

**Authors:** Christos Iavazzo, George Vorgias, Paraskevi-Evangelia Iavazzo, Ioannis D. Gkegkes

**Affiliations:** 1 Department of Gynecological Oncology, Christie Hospital, Manchester, United Kingdom; 2 Department of Gynecological Oncology, Metaxa Cancer Hospital, Piraeus, Greece; 3 Department of Pediatrics, Rethymno Hospital, Rethymno, Crete, Greece; 4 First Department of Surgery, General Hospital of Attica “KAT”, Athens, Greece

**Keywords:** Immature teratoma, ovarian germ cell tumor, fertility-sparing surgery, premenopausal, treatment

## Abstract

Immature teratomas are quite rare tumors arising in young women. They are usually diagnosed in early stage and grade and have a good prognosis. In these young patients, fertility-sparing management is suggested as the standard of care. Bilateral immature teratoma is a rare condition with an incidence of 10%, with a five-year survival rate of 80%. The majority of patients received fertility-sparing treatment followed by adjuvant chemotherapy in 78%. Older age, advanced stage, and high grade are negative prognostic factors. The surgery-only, watch-and-wait approach was evaluated; however, after a median follow-up time of 42 months, 50% of patients experienced recurrence, but they were successfully salvaged with chemotherapy. In a retrospective study, 12 out of 27 patients tried to conceive, resulting in 10 pregnancies (8 after chemotherapy). We present a narrative review of the current literature regarding the essential multidisciplinary approach of such patients in order to achieve the best oncologic and fertility-sparing outcome.

## INTRODUCTION

The term teratoma has its origin from the Greek word “teras” which means monster (1). The term began to be used about 300 years ago because people believed that these types of tumor were either aborted embryos or embryonic structures (2). Teratoma represents one of the most frequent germ cell neoplasms, which contain elements of all three germ cell layers (3). This type of neoplasm arises from totipotent cells in the ovary, which develop progressively into ectodermal, mesodermal, and endodermal tissue (4). Teratomas account for 10 to 20% of all ovarian malignancies in women younger than 20 years (5, 6).

There is a grading system based on the proportion of mature and immature neuroepithelial tissues, mitotic activity, and degree of differentiation. The following grades are proposed: grade 0= tumors with only mature tissue, and grades 1, 2, 3 all with mitotic activity, but with limited, moderate or large amounts of immature neuroepithelial tissue, respectively. Surgical management of teratomas is based on the following criteria: symptomatic relief, tumor size (often defined as greater than 5 cm in diameter given the propensity for torsion), and malignant potential (7). Immature teratomas are less common and definitely more aggressive tumors (8). The “immature” character of these tumors and the presence of not fully differentiated cells reveal a more aggressive behavior as the 30% of the ovarian cancer mortalities between the ages of 10 and 20 years is attributed to immature teratomas (4). As a consequence, the selection of proper treatment is fundamental because its priorities should include an oncologic approach to achieve the best possible prognosis by minimizing the risk of recurrence while considering the option of preserving the patients’ future fertility.

The aim of this narrative review was to clarify the role of fertility-sparing surgery as a treatment option for young patients with immature teratomas based on the available current literature.

## METHODS

An extensive electronic search was performed in PubMed (27/08/2015) and Scopus (27/08/2015). The adopted search strategy included the combination of the following keywords: treatment and fertility and (immature teratoma or immature ovarian teratoma). In order to retrieve additional studies, the references of the included studies were also searched. Studies written in languages other than English were not included. The literature search had a limitation at the search range, only studies written after 1990 were considered eligible for this review. Nine studies were eligible to be included in our review. Studies reporting data on fertility-sparing surgery in premenopausal patients with immature teratoma were regarded as eligible for this review. Abstracts, conference papers, book chapters, animal studies, commentaries, editorials as well as review articles were excluded from this review.

## DISCUSSION

Immature teratomas are usually unilateral tumors, which can be associated with the presence of ascites. They are usually curable at an early stage. The prognosis is correlated with the grade of immature elements. Stage Ia grade 1 tumors have an adequately good prognosis (94%) if treated surgically with complete staging procedures and are excised unruptured (9). Higher grade tumors have a survival of 85% (9, 10). The need for adjuvant chemotherapy in stage Ia G2-G3 and Ib-Ic is still controversial. Bilateral immature teratoma is a rare condition with an incidence of 10% and a five-year survival rate of 80% (11, 12). According to a recent study that included 27 patients with immature ovarian teratoma, the median age at diagnosis was 27.0 years (range, 18-36 years). Eighty-two percent presented with stage I disease, 11% had stage II, and 7% had stage III disease. Thirty-three percent of the patients were grade 1, 11% grade 2, and 56% grade 3. The majority of the patients received fertility-sparing treatment followed by adjuvant chemotherapy in 78% (13). Jorge et al. (14), in a recent multivariate analysis, showed that 90% of patients with immature teratoma were aged 18 to 39 years, three quarters of whom were early staged and could be treated conservatively combining fertility-sparing surgery and chemotherapy. Older age, advanced stage, and high grade are negative prognostic factors (14).

Fertility preservation in the cancer setting, known as oncofertility, became one of the hot topics in patients with cancer. An Oncofertility Consortium funded by the National Institute of Health has been established since 2006 and there are currently 19 countries engaged in the global oncofertility community. Medical awareness should be raised in young patients with ovarian tumors such as immature teratomas regarding fertility preservation options (15, 16). The main aims of such a multicenter international approach would be to standardize and improve the quality of treatment for every patient in each institute, to make a referral system that will further clarify outcomes and prognosis for the oncofertility approach of such rare tumors, and provide a policy to support each hospital (15).

**Figure 1:**
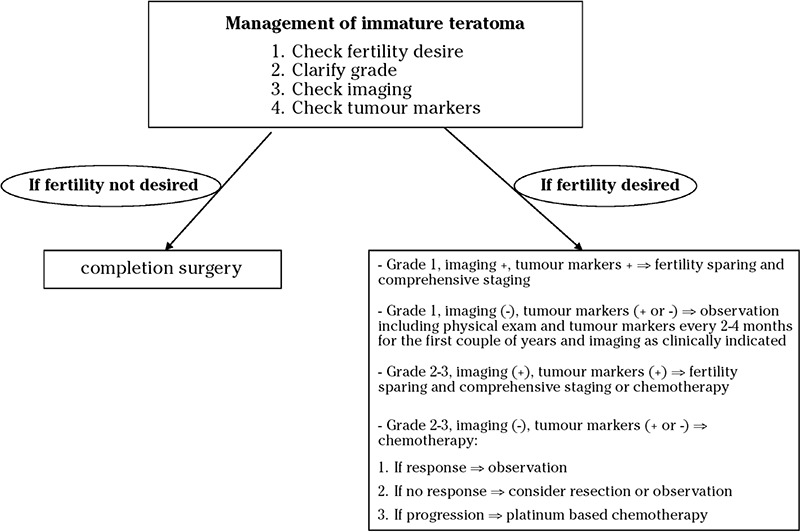
Algorithm on the management of patients with immature teratoma (adopted from National Comprehensive Cancer Network guidelines 2015)

Based on the good prognosis of patients with early-stage immature teratoma, fertility-sparing surgery, which could include removal of the affected ovary and preserving the contralateral ovary and uterus, followed by combination chemotherapy has become the standard of care of early-stages immature teratomas (10). Figure 1 presents an algorithm on the management of patients with immature teratoma, based on the National Comprehensive Cancer Network guidelines (17). The surgical treatment of ovarian teratomas should be based on ultrasound diagnosis regarding tumor size and possible extra ovarian findings, and a laparoscopic fertility-preserving approach is recommended (18). Specifically, based on the surgeon’s experience and the size of the tumor >10 cm or not, a decision can be reached regarding the open or minimal invasive approach (19). After informed consent, open, laparoscopic or a robotic approach can be used in these patients with the aim of complete cytoreduction. More specifically, unilateral salpingo-oophorectomy with surgical staging is suggested including peritoneal exploration, cytology, and biopsies, omental biopsy or omentectomy and pelvic and/or para-aortic lymph node dissection. During surgery, routine biopsy of the normal-appearing contralateral ovary should be avoided because biopsy of the contralateral ovary could lead to future infertility related to peritoneal adhesions or ovarian failure (20). Park et al. (21) showed in their retrospective study that pelvic, para-aortic lymph node dissection, omentectomy and appendectomy was performed in only 36%, 26%, 59%, and 22%, respectively. Naturally, the role of systematic pelvic and para-aortic lymph node dissection could be questioned by several groups especially in patients with early-stage immature teratomas. The widespread use of a laparoscopic bag decreased the incidence of tumor spillage into the peritoneal cavity (22). However, minimal invasive techniques are characterized by increased operating times, increased cost, but also less postoperative pain, fewer adverse events of surgery, and a shorter length of stay in hospital (23, 24). Moreover, Chatchotikawong et al. (25) showed in an eight-year analysis that bilateral pelvic and paraaortic lymphadenectomy could offer information regarding disease extension and prognosis, elimination of some microscopic tumors, and clarify the further treatment options postoperatively. As Brown et al. (26) mentioned the lack of full surgical staging could lead to limited tissue evaluation and inappropriate over- or under-treatment options. Additionally, the MITO-9 study showed that in cases of bilateral immature teratomas, although bilateral salpingo-oophorectomy could be performed, enucleation of contralateral tumor could be a treatment option based on their high chemosensitivity (27). Furthermore, Beiner et al. (28) showed that 5 out of 8 patients with bilateral immature teratoma who had conservative management received adjuvant chemotherapy, but all were disease free after 5 years’ follow-up.

Regarding the role of chemotherapy, it is known that there is no need for adjuvant treatment in stage Ia grade 1 immature teratomas, whereas bleomycin, etoposide, and cisplatin (BEP) are used with excellent results in later stages and/or grades (6). More specifically, this surgery-only, watch-and-wait approach was evaluated; however, after a median follow-up time of 42 months, 50% of patients experienced recurrence, but they were successfully salvaged with chemotherapy (29). In addition, Park et al. (30) showed that fertility-sparing surgery alone with surveillance could be a safe treatment strategy in such patients and the majority of recurrences could be salvaged surgically and using BEP chemotherapy. Excessive chemotherapy may be harmful for the function of the preserved ovary. The reported histologic changes in the ovaries of patients receiving chemotherapy include cortical fibrosis, reduction in number of follicles, and impaired follicular maturation. These changes may lead to hypogonadism (31, 32). Post-operative radiotherapy has not proven to be of benefit (33).

Prognosis is excellent in early-stage and low-grade tumors. Hannan et al. (34) showed that all recurrences were found in patients advanced-stage tumors with recurrence rates reaching 13% in such patients. Fertility-sparing surgery should be attempted whenever possible under the care of subspecialists and after a thorough multidisciplinary decision and informed consent of the patients and/or her parents. Advice should be also taken from specialists in subfertility prior to surgery in cases of bilateral ovarian involvement in order to discuss the possibility of uterine conservation for possible future pregnancy with frozen or donor eggs. An international database that includes patients who have undergone fertility-sparing treatment could further clarify the prognosis, recurrence rates, reproductive and fertility outcomes, as well as the possible alternative treatment options in patients with recurrence. The first report of a successful pregnancy following conservative surgery and chemotherapy for advanced-stage immature teratoma appeared in 1989 (35). To date, several studies have shown that successful pregnancy is possible after treatment for immature teratoma, despite the administration of chemotherapy and surgery (35, 36). Alwazzan et al. (13) showed in their retrospective study that 12 out of 27 patients tried to conceive resulting in 10 pregnancies (8 after chemotherapy). Long-term follow-up of patients with immature teratoma treated conservatively is very important to clarify recurrence rates and salvage options.

## CONCLUSION

Fertility-sparing surgery could be offered to young patients with immature teratomas especially at early stage and grade. A multidisciplinary approach towards the patient including gynecologic oncologists, subfertility specialists, medical oncologists, and psychologists is suggested to optimize the care offered.
